# Marginal Bone Loss around Implant-Retaining Overdentures versus Implant-Supported Fixed Prostheses 12-Month Follow-Up: A Retrospective Study

**DOI:** 10.3390/ijerph19031750

**Published:** 2022-02-03

**Authors:** Odontuya Dorj, Chin-Kai Lin, Eisner Salamanca, Yu-Hwa Pan, Yi-Fan Wu, Yung-Szu Hsu, Jerry C.-Y. Lin, Hsi-Kuei Lin, Wei-Jen Chang

**Affiliations:** 1 School of Dentistry, College of Oral Medicine, Taipei Medical University, Taipei 11031, Taiwan; dorj.odontuya@gmail.com (O.D.); 01728@km.eck.org.tw (C.-K.L.); eisnergab@tmu.edu.tw (E.S.); shalom.dc@msa.hinet.net (Y.-H.P.); yfwu@tmu.edu.tw (Y.-F.W.); nm8346@yahoo.com.tw (Y.-S.H.); drjerrylin@gmail.com (J.C.-Y.L.); 2 Department of Dental Technology and Dental Hygiene, School of Dentistry, Mongolian National University of Medical Sciences, Ulaanbaatar 14210, Mongolia; 3 Department of Dentistry, En Chu Kong Hospital, New Taipei City 237, Taiwan; 4 Department of Dentistry, Chang Gung Memorial Hospital, Taipei 105406, Taiwan; 5 Graduate Institute of Dental & Craniofacial Science, Chang Gung University, Taoyuan 33305, Taiwan; 6 School of Dentistry, College of Medicine, China Medical University, Taichung 40402, Taiwan; 7 Department of Oral Medicine, Infection and Immunity, Harvard School of Dental Medicine, Boston, MA 02115, USA; 8 School of Oral Hygiene, College of Oral Medicine, Taipei Medical University, Taipei 11031, Taiwan; 9 Dental Department, Taipei Medical University, Shuang-Ho Hospital, New Taipei City 23561, Taiwan

**Keywords:** non-submerged dental implant, marginal bone loss, radiographic bone-implant interface contact, implant-retained overdenture, implant-supported fixed prosthesis

## Abstract

Few studies have compared marginal bone loss (MBL) around implant-retaining overdentures (IODs) vs. implant-supported fixed prostheses (FPs). This study evaluated the mean MBL and radiographic bone-implant interface contact (r-BIIC) around IODs and implant-supported FPs. We also investigated osseointegration and MBL around non-submerged dental implants. We measured the changes between the MBL in the mesial and distal sites immediately after prosthetic delivery and after one year. The mean MBL and its changes in the IOD group were significantly higher. The mean percentage of r-BIIC was significantly higher in the FP group. MBL and its changes in males were significantly higher in the IOD group. The percentage of r-BIIC was significantly higher in the FP group. MBL in the lower site in the IOD group was significantly higher. Regarding MBL, the location of the implant was the only significant factor in the IOD group, while gender was the only significant predictor in the FP group. Regarding the r-BIIC percentage, gender was a significant factor in the FP group. We concluded that non-submerged dental implants restored with FPs and IODs maintained stable bone remodeling one year after prosthetic delivery.

## 1. Introduction

The introduction of osseointegrated dental implants that support a variety of prostheses has been a breakthrough in oral rehabilitation. There are various attachments; however, edentulous patients are considerably more satisfied with two implant-retained overdentures (IODs) than with complete dentures [1]. IODs demonstrate better retention and fit and improved function and quality of life compared to complete dentures. According to Gotfredsen et al., the success rate of IODs was 100 percent [2]. Recently, on the other hand, a newly designed prosthetic modality, i.e., implant-crown-retained removal partial dentures (IC-RPDs), was proposed in a retrospective study [3]. They suggested IC-RPDs as a feasible treatment option for edentulous patients. According to Kang et al., the survival rate of IC-RPDs for partially edentulous mandibular arches was 93.1% [4]. The survival rate of IC-RPDs was 98.3%, while the survival rate of IODs was 92.6%, which was not significantly different [3]. Selim et al. concluded that implant-supported fixed prostheses (FPs) in the mandibula showed better outcomes regarding patient satisfaction. On the other hand, higher satisfaction scores were found for IODs in the maxilla than implant-supported FPs [5].

A recent retrospective study demonstrated marginal bone resorption around implants in the two and three implant-supported partial FPs in the posterior region of the mandible. This study revealed that mesial implants had more marginal bone loss (MBL) than distal implants. The parameters including smoking habits, restoration types and fractures; parafunctional activity did not affect MBL in the two and three implant-supported FP groups [6]. Evaluating clinical and radiographic measurements regarding dental implants and their substructures is crucial to resolving the determinants of treatment successes and failures. A review study evaluated the MBL around implant-supported single FPs and multiple-unit screw-retained prostheses. The mean MBL was 0.58 mm and 0.90 mm for the single and multiple-unit prostheses, respectively, with no statistical difference [7]. A meta-analysis demonstrated a mean MBL of 0.53 mm for cement-retained prostheses and 0.89 mm for screw-retained prostheses with no statistical difference [8]. The one-year MBL for removable prostheses varied from 0.13 ± 0.35 mm to 1.03 ± 0.65 mm, whereas the MBL for FPs varied from 0.05 ± 0.67 to 1.37 ± 0.5 mm after one-year restoration [9].

Another essential prerequisite for dental implant success is osseointegration, the percentage of bone that can determine implant interface. Several studies have suggested that 50–80% bone to implant contact occurred in clinically successful implants [10,11]. Buser et al. prospectively studied non-submerged dental implants restored with removable and fixed restorations. They found more favorable success rates for screw-type implants than hollow-cylinder implants (95% and 91.3%, respectively). In addition, they demonstrated better success rates for mandibular implants compared with maxillary implants (95% and 87%, respectively) [12]. XB Duan et al. identified the relationship between MBL and microbiota in the saliva. MBL severity was linked to increased amounts of pathogenic periodontal species; thus, the authors suggested a crucial role for the microbiome in the progression of MBL during the bone healing period [13].

In addition, the MBL around the implants is also considered one of the criteria for outcome evaluation. The criteria of dental implant success proposed by Albrektsson et al. included immobility, no evidence of peri-implant radiolucency, absence of pain and inflammation, and 0.2 mm annual crestal bone loss following the one-year loading of implants [14]. A large-scale study of 1673 implants revealed that 3-unit FDs with one pontic had significantly more marginal crestal bone loss than single crown-supported implants one year after loading [15]. The systematic review of Zimmermann et al. revealed comparable MBL around dental implants restored with fixed and removable prostheses after one year [9]. Another recent systemic review reported that implant-supported fixed and removable prostheses appeared to have homogenous long-term MBL [16]. However, the limited number of related studies pointed to the necessity for well-designed studies comparing the treatment modalities mentioned above. The lack of clinical trials that compared the MBL around an implant-retained removable prosthesis versus FPs was emphasized in recent systemic reviews [9,16].

Hence, the present study aimed to evaluate the outcome of the mean MBL and the radiographic bone-implant interface contact around IODs and implant-supported FPs.

## 2. Materials and Methods

### 2.1. Data Collection

A total of 60 non-submerged ITI implants (Straumann AG, Waldenburg, Switzerland) were placed in 46 patients. These patients consisted of 22 males and 24 females aged between 30–83 years with a mean age of 54.41 ± 1.93 years. According to our clinical report, inclusion criteria were: (1) patients who treated with IODs or implant-supported FPs; (2) patients who received one or more ITI implants; (3) no active inflammation around implant site; (4) keratinized tissue more than 2 mm at the time of implant installment. Patients with systematic diseases, tobacco smoking and betel nut chewing habits and a membrane or a bone graft, poor oral hygiene, lost to follow-up, and breastfeeding or pregnant, long-term oral-medicated patients were excluded. The study samples were grouped into two categories; i.e., either restored with IODs or implant-supported FPs. The study protocol was approved by the Institutional Review Board of Taipei Medical University (N202103105). Data were collected from Shuang-Ho Hospital, Taipei Medical University. The most frequent location for implant placement was the lower jaw (70%). All 60 implants in this study were standard soft tissue level implants; 29 had a diameter of 4.1 mm (length of 10 mm), 25 had a diameter of 4.8 mm (with lengths of 8 mm or 10 mm) and six had a diameter of 3.3. mm (with a length of 10 mm).

### 2.2. MBL and r-BIIC Measurements

**Figure 1:**
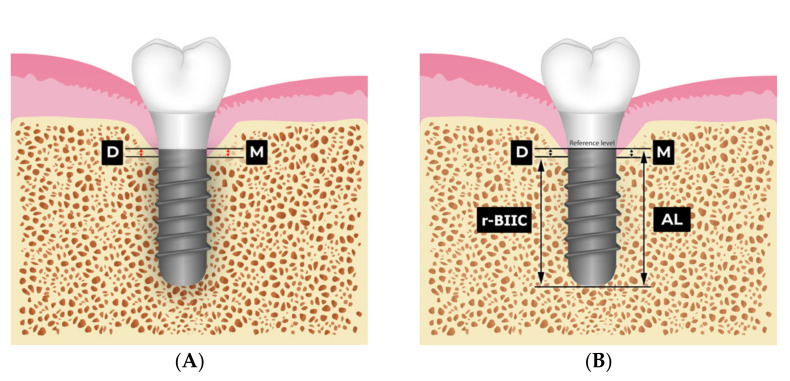
Vector illustration of the non-submerged dental implants (ITI Straumann implant). (**A**) MBL at mesial (M) and distal (D) sites. (**B**) AL, actual length of the implant; r-BIIC, radiographic bone-implant interface contact.

A number of variables were collected from medical records and radiographs of the patients immediately after prosthetic delivery (baseline) and at the one-year follow-up visit. Digital panoramic X-ray equipment Hyper-X CM (Asahi Roentgen IND, Kyoto, Japan) was utilized. The paralleling technique and X-ray cone indicator were used for all standard periapical radiographs and patients were informed to bite on the film. The images were taken immediately after prosthetic delivery and one-year after loading. Bone levels were measured from the smooth–rough surface of the non-submerged implants to the alveolar crest on the mesial and distal sites (Figure 1).

We defined MBL as the alteration between the mean value of the marginal bone loss in the mesial and distal sites immediately after prosthetic delivery and at the one-year follow-up visit (Figure 1A). For the percentage of r-BIIC, the measurement was calculated by subtracting the actual length (AL) of the implant and changes in MBL divided by the AL (Figure 1B). The proportion of the bone-implant interface contact of the implants was determined using the osseointegrated part and the bone loss. We denoted radiographic bone-implant interface contact as r-BIIC in the study. The percentage of the bone was calculated from length of the dental implant and the vertical marginal bone loss.

All radiographs were registered and accumulated by two examiners and the dental surgeon who performed the implant installation. Professional dental image software was utilized to determine all measurements (EZ dental software, Asahi Co., Ltd., Tokyo, Japan). The length calibration tool of the EZ dental professional image software was used to correct the digression of the periapical film. The definite length of the dental implant was used to adjust the periapical film. Subsequently, an adjusted length measuring tool was used to derive the MBL in the mesial and distal sites of the implant. In this study, we detected marginal bone level (MBL), its change (MBLC) and radiographic bone-implant interface contact (r-BIIC) around implants based on various variables including gender, location and the diameter and length of the placed implants.

### 2.3. Statistical Methods

Statistical analyses and the creation of graphs were performed using GraphPad Prism software version 8.0 for Mac (GraphPad Software, San Diego, CA, USA). To examine whether the obtained data were normally distributed, Shapiro–Wilk normality tests were applied. All data were presented as the mean and standard errors. A paired *t*-test was applied to evaluate the differences in MBL, its change and the r-BIIC within groups. An independent *t*-test with Welch’s correction was used to examine the aforementioned variables between the IOD and FP groups. The effect of implant parameters on MBL, changes in MBL, and the r-BIIC in IOD and FP groups were determined with an unpaired *t*-test with Welch’s correction and Welch’s ANOVA test. For all statistical tests, the significance level was set at *p* < 0.05.

## 3. Results

### 3.1. Demographic Data and Implant Distribution

A total of 46 patients (22 males and 24 females) were included in the study. The average age of the patients was 54.4 ± 1.93 years at the time of implant placement. The demographic data and implant characteristics are presented in Table 1. Most patients (74%) received one implant and 26% received two or more implants. Single tooth implants were included in all FP cases and sixteen arches were IOD treatments.

### 3.2. Marginal Bone Loss Assessment

**Figure 2:**
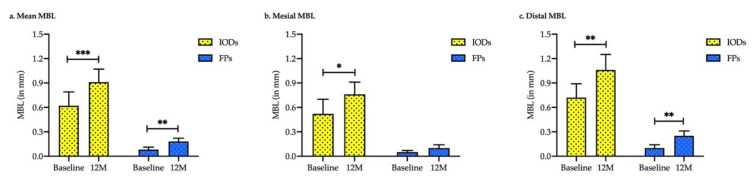
Marginal bone level alteration at baseline and 12-month follow-up after prosthetic delivery of implant-supported fixed protheses (FPs) and implant-retaining overdentures (IODs). (**a**) Mean MBL; (**b**) MBL in mesial site; and (**c**) MBL in distal site. A paired *t*-test was used. *** *p* < 0.001, ** *p* < 0.01 and * *p* < 0.05.

Figure 2 and Table 2 show the results from an analysis of MBL around the non-submerged implants in the IOD and FP groups. The mean MBL at 12 months was 0.91 ± 0.16 mm for the IOD group and 0.18 ± 0.04 mm for the FP group. The mean MBL of non-submerged implants in the IOD and FP groups was statistically significant (baseline vs. 12 months; *p* < 0.001). The MBL of the mesial sites of implants in the FP group did not differ from the baseline or at 12 months follow-up, while the MBL in the IOD group was significant (*p* < 0.017). However, the MBL at the distal sites of implants was statistically significantly different in the IOD and FP groups (*p* < 0.01).

**Figure 3:**
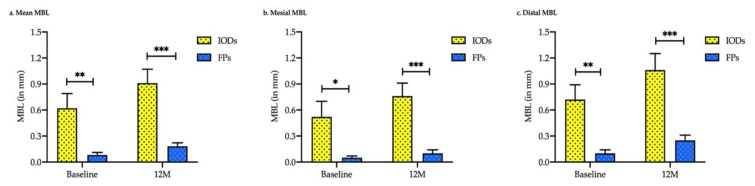
Marginal bone loss comparison between implant-supported fixed prostheses (FPs) and implant-retaining overdentures (IODs) at different time points. (**a**) Mean MBL; (**b**) MBL at the mesial site; and (**c**) MBL at the distal site. An unpaired *t*-test with Welch’s correction was used. *** *p* < 0.001, ** *p* < 0.01, and * *p* < 0.05.

Furthermore, the mean MBL between groups was significantly different at baseline (*p* = 0.003) and at 12 months (*p* < 0.001). A similar trend of MBL was observed in the mesial and distal sites of non-submerged dental implants. The mean mesial MBL was statistically significant between the IODs and FP groups at baseline and at 12 months follow-up (*p* = 0.016 and *p* < 0.001, respectively). Comparably, the mean distal MBL was significant at baseline and at 12 months follow-up (*p* = 0.001 and *p* < 0.001, respectively; Figure 3 and Table 3).

**Figure 4:**
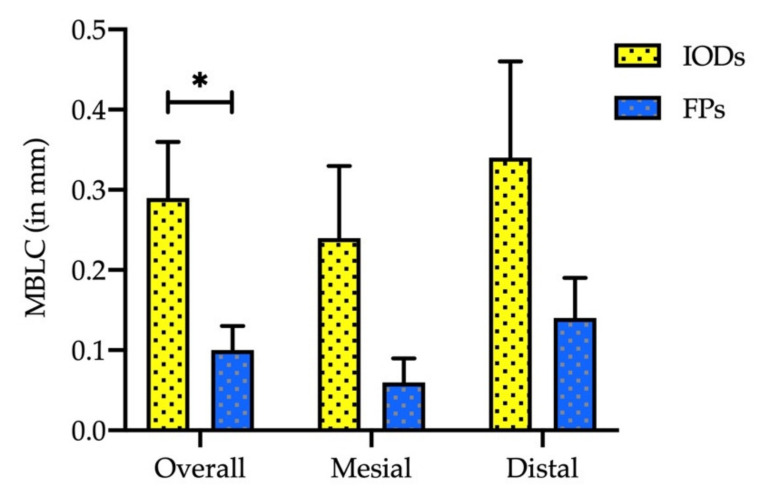
Change in marginal bone loss comparison between implant-supported fixed protheses (FPs) and implant-retaining overdentures (IODs) after 12 months prosthetic delivery. An unpaired *t*-test was used. * *p* < 0.05.

Figure 4 exhibits the changes in MBL between the IOD and FP groups. Overall, the changes in MBL were significantly higher in the IOD group (IOD group: 0.29 ± 0.07 mm; FP group: 0.10 ± 0.03 mm; *p* = 0.018), whereas the mesial and distal MBL did not reveal any significance.

### 3.3. Bone-Implant Interface Contact Analysis

**Figure 5:**
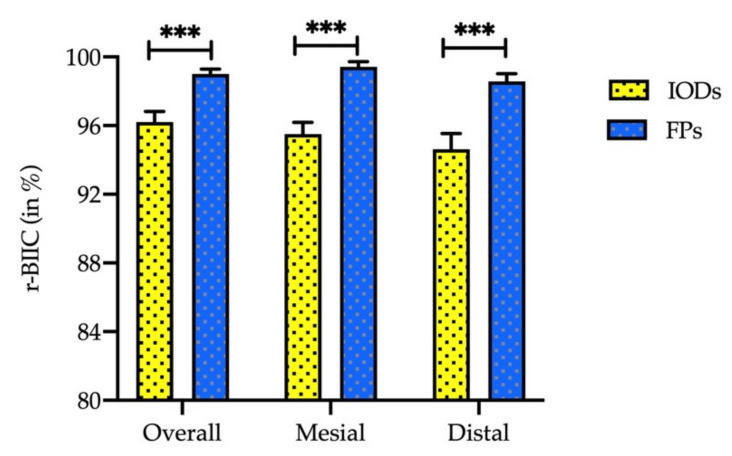
Radiographic bone-implant interface contact (r-BIIC) percentage comparison between the implant-supported fixed prostheses (FP) group and the implant-retained overdentures (IOD) group at 12 months after the prosthetic delivery. *** *p* < 0.001.

A radiographic bone-implant interface contact (%) analysis was carried out (Figure 5). The mean r-BIIC percentage was significantly higher in the FP group than in the IOD group (*p* < 0.001). Likewise, the mesial and distal r-BIIC percentages were significantly higher in the FP group than in the IOD group (*p* < 0.001).

### 3.4. The Effect of Implant Parameters According to Gender, Diameter and Length of the Implant

The effect of gender on MBL and osseointegration outcomes is presented in Table 4. Interestingly, statistically significant differences in MBL, changes in MBL and the r-BIIC percentage were observed in the male group only, whereas no significance was found in the female group. MBL in the male group was significantly higher in the IOD group than in the FP group (0.91 ± 0.12 mm and 0.03 ± 0.02 mm, respectively; *p* < 0.001), while changes in MBL were statistically significantly different between the two groups (0.34 ± 0.11 mm in the IOD group and 0.03 ± 0.02 mm in the FP group, respectively; *p* < 0.05). Similarly, the r-BIIC percentage in the male group was higher in the FP group than in the IOD group (95.40 ± 0.99% and 99.75 ± 0.25%, respectively; *p* < 0.001).

Table 5 shows the effect of implant location on MBL and osseointegration outcomes. MBL in the lower jaw was significantly higher in the IOD group than in the FP group (1.16 ± 0.14 mm and 0.13 ± 0.04 mm, respectively; *p* < 0.001), while the change in MBL was not statistically significantly different between the two groups (0.28 ± 0.09 mm in the IOD group and 0.12 ± 0.04 mm in the FP group, respectively; *p* < 0.115). Unlike the above results, the r-BIIC percentage was statistically significantly different and higher in the FP group than in the IOD group (*p* < 0.001).

Moreover, Table 6 presents the effect of implant length on MBL and osseointegration outcomes. MBL for implants with a length of 10 mm was significantly higher in the IOD group than the FP group (00.90 ± 0.17 mm, and 0.18 ± 0.04 mm, respectively, *p* < 0.001), whereas changes in MBL in implants with a length of 10 mm were statistically significantly different between the two groups (*p* < 0.01). The r-BIIC percentages in implants with a length of 10 mm were statistically significantly different between the two groups and higher in the FP group (*p* < 0.001).

### 3.5. The Effect of Implant Parameters on Osseointegration and MBL Outcomes

The effect of patient and implant parameters on osseointegration and MBL outcomes at the 12-month follow-up in the IOD group is shown in Table 7. Genders of the patients, location, diameter and length of the implants were analyzed in the IOD group. Considering implant parameters, the implant location was the only significant factor that affected the MBL around non-submerged dental implants. MBL in the lower jaw was higher than in the upper jaw in the IOD group analysis (0.09 ± 0.41 mm and 1.16 ± 0.14 mm, respectively; *p* = 0.042). All other parameters did not reveal any significance in the IOD group.

The effect of patient and implant parameters on osseointegration and MBL outcomes at the 12-month follow-up in the FP group is shown in Table 8. Genders of the patients, location and diameter of the implants were analyzed in the FP group. The gender of the patient was the sole factor that significantly affected the MBL, change in MBL and r-BIIC percentage around non-submerged dental implants. The MBL in the male group was significantly lower than in the female group in the FP group (0.03 ± 0.03 mm and 0.28 ± 0.06 mm, respectively; *p* = 0.002). Similarly, MBL changes in males were significantly lower than in the female group (0.03 ± 0.03 mm and 0.15 ± 0.04 mm, respectively; *p* = 0.008). The r-BIIC percentage in the male group was significantly higher than in the female group (99.75 ± 0.25% and 98.50 ± 0.42%, respectively; *p* = 0.008). The location and diameter of the implants did not reveal any significance in the IOD group.

## 4. Discussion

A previous study reported that MBL averaged between 1.5 mm to 2.0 mm in the first year, and 0.2 mm annual bone loss was observed [14]. In the present study, after one-year prosthetic loading, the mean MBL around the implant-supported FPs was 0.18 ± 0.04 mm, while in the implant-retained IOD group, the mean MBL was 0.91 ± 0.16 mm. Given the fact that the implant-supported restorations revealed MBL within the scope of the implant success criteria documented by the researchers [14], the implant-supported IODs had significantly more bone loss after loading for one year. Thus, the null hypothesis was supported that implant-retained IODs may be more prone to bone loss after one-year prosthetic loading.

A 20-year retrospective study reported a greater than 95% overall survival rate for dental implants with overdentures, and this is a clinically accepted routine treatment for an edentulous mandible [17]. Another study revealed that the survival rate was 95.9% for implant IODs after a 75-month recall, and the corresponding MBL was 0.86 ± 0.92 mm one year after loading [3]. This 1-year result for the MBL level corresponded with our study. Additionally, the authors did not find a higher MBL for implants in the IOD group than in the FP group, which contradicts the results of our study. These may be due to different implant systems and a more extended examination period. After six months of observation, the MBL of implants in the IOD group was 1.99 ± 0.70 mm [18]. Again, this result was inconsistent with our study.

The MBL around implant-retained mandibular IODs was not affected by age or gender [19]. Similarly, the diameter and the length of the implants did not affect MBL and its changes in our study. However, our study showed a significant difference in the MBL of implants regarding the location (upper jaw vs. lower jaw). Another clinical study revealed that peri-implantitis at the one-year visit affected the MBL around implants; therefore, the pathological changes need to be scrutinized to prevent severe MBL problems [3]. The placement location of the implants is complex, and it should be determined carefully, considering the force and possibility of changing the restoration. IODs with anteriorly positioned implants could be a viable treatment option clinically, particularly for patients with severe absorption in the posterior region. The observation period was only one year; therefore, further prospective studies with longer follow-up times are necessary.

By definition, FPs are cemented or screwed abutment connections to the implant frame. Reported after one year, MBL was over 1 mm in screw-retained fixed implant-supported FPs [20]. Several studies examined the contradicting MBL differences between screw-retained and cemented fixed implant restorations. Higher MBL were observed in screw-retained FPs [21,22,23], while cement-retained FPs had greater MBL around the implants than screw-retained restorations [24,25]. In a systematic review, the MBL between a screw and cement-retained implant-supported restoration was not significantly different, and the findings were not consistent with previous studies [8,26]. A systematic review of MBL around implant-supported fixed vs. removable prostheses reported that MBL in the first year ranged from 0.17 ± 0.07 mm to 2.1 ± 1.6 mm for fixed implant-supported prostheses; these results were compatible with the present study. Regarding the MBL, fixed and removable implant-supported prostheses appeared to have comparable long-term results. They suggested that related research designs are necessary to contribute detailed information about MBL changes [16].

Zimmermann et al. studied MBL at 1-year after implant placement using intraoral periapical radiographs, and the observation period was similar to the present study. Of the 22 selected studies, the minimum MBL was 0.05 ± 0.67 mm and the maximum MBL was 1.37 ± 0.50 mm for the fixed restorations, while the MBL for removable restorations varied from 0.13 ± 0.35 mm to 1.03 ± 0.65 mm. The difference in MBL between the restorations, as mentioned earlier, was 0.36 mm in this review. The authors concluded that dental implants restored with fixed and removable prostheses showed equivalent MBL at one year after implant placement [9]. The outcomes of our study were comparable to these results, showing an MBL of 0.91 ± 0.16 mm for the IOD group and 0.18 ± 0.04 mm for the FP group. Park et al. performed randomized clinical trials comparing two different non-submerged dental implants; the test group received the Osstem SSII Implant, and the control group received the Standard Straumann Dental Implant. After a one-year follow-up, the mean MBL was 1.07 ± 0.46 mm for the control group and 0.79 ± 0.42 mm for the test group [27]. The MBL difference was minimal between these findings and the present study. However, the observation period differed from the previous study; they examined the MBL one year after implantation. We evaluated the MBL one year after prosthetic delivery, which may have caused the difference in the MBL range.

Recently, researchers measured the r-BIIC using three-dimensional imaging. The mean r-BIIC length was longer in males than females (*p* = 0.028), and the r-BIIC length in zygomatic implants was longer than in single implants (*p* = 0.027), which suggested that zygomatic implants are a feasible treatment option in complete mouth restoration [28]. In contrast, our study presented the percentage of r-BIIC between two implant-supported restorations: 96.20 ± 0.62% for the IOD group and 99.0 ± 0.29% for the FP group, with a statistically significant difference between the two groups. Furthermore, the degree of MBL was due to implant-related mechanical factors and biological factors, such as occlusal overload, peri-implantitis, micro-gap, gender, age, implant position and implant restorations. Ozgur et al. evaluated the effect of implant brand, location, width and length of the implant and smoking on MBL in a 6-year observation period. The MBL was affected by the location of the implant; it was more significant in the posterior maxillary area [29]. This result was inconsistent with our findings of higher MBL in implants with IODs in the posterior mandible area; however, it was consistent with higher MBL in FPs in the maxillary posterior region. Single implant-supported FPs showed lower MBL than multiple-unit FPs due to the inter-proximal hygiene being much more complicated around multiple-unit restorations, which is still not completely understood [30]. Moreover, age and gender are important factors in bone preservation, and bone mass density declines with age. Females lost more bone than males in the FP group in the present study, whereas MBL was comparable across gender in the IOD group. A clinical study found the highest MBL in a female group [31,32]. This outcome could be related to the physiological changes in older women that can profoundly impact bone loss.

Nevertheless, there are limitations to this study that need to be stated. The major limitation of this study was its retrospective design and the low precision of the patients’ medical records. In addition, the present study had limitations that included a lack of information about the opposing teeth structure and the soft tissue thickness, a short follow-up period, different diameter and length of the placed implants and limited details of the position of the examined implants. Furthermore, examining the MBL around implants with a two-dimensional structure only enabled the assessment of the mesial and distal aspects of the placed implant. Therefore, further studies are necessary to confirm the results and consider these limitations in the future.

## 5. Conclusions

Statistically significant stable bone remodeling was observed in both the IOD and FP groups. The mean MBL was significantly greater in the IODs compared to the FPs. Thus, the percentage of r-BIIC was notably higher in the FP group. In terms of the location of the implant, MBL in the lower site was significantly higher in the IOD group than the FP group, while the r-BIIC percentage was higher in the FP group than in the IOD group. Regarding the effect on MBL, the location of the implant was the sole significant factor in the IOD group. Likewise, regarding the effect on the MBL and the alteration in the MBL, gender was the only predictor in the FP group. Therefore, within the limitations of the present study, it can be concluded that non-submerged dental implants restored with implant-supported FPs and the implant-retained IODs maintain stable bone remodeling one year after prosthetic delivery. In addition, MBL in the male group was significantly less in the FP group than in the IOD group.

## Figures and Tables

**Table 1:** Demographic data and implant related information.

**Characteristics**	**Total**		**IOD Group**		**FP Group**	
	***n* = 46**		***n* = 16**		***n* = 30**	
Age, mean (SEM)			66.31 (2.54)		48.07 (1.75)	
Gender (*n*, %)						
Male	22	47.8%	10	62.5%	12	40.0%
Female	24	52.2%	6	37.5%	18	60.0%
**Implant Parameters**	***n* = 60**		***n* = 30**		***n* = 30**	
Location (*n*, %)						
Upper jaw	18	30.0%	7	23.3%	11	36.7%
Lower jaw	42	70.0%	23	76.7%	19	63.3%
Diameter (*n*, %)						
3.3 mm	6	10.0%	6	20.0%	0	0.0%
4.1 mm	29	48.3%	22	73.3%	7	23.3%
4.8 mm	25	41.7%	2	6.7%	23	76.7%
Length (*n*, %)						
10 mm	58	96.7%	28	93.3%	30	100.0%
8 mm	2	3.3%	2	6.7%	0	0.0%

**Note:** IOD, implant-retaining overdentures; FP, implant-supported fixed prostheses. The table includes a total of 46 patients and 60 implants (For IODs, 11 patients received two implants, 1 four implants, and 4 one implant (they had one implant in the past), while 30 patients received 30 implants in the FP group).

**Table 2:** Marginal bone level alteration: Intra-group analysis of IODs and FPs.

Time Period	IOD Group	FP Group
Average		
Baseline	0.62 ± 0.17	0.08 ± 0.03
12-month	0.91 ± 0.16	0.18 ± 0.04
*p*	<0.001 ***	<0.002 **
Mesial site		
Baseline	0.52 ± 0.19	0.05 ± 0.02
12-month	0.76 ± 0.15	0.10 ± 0.04
*p*	0.017 *	0.061
Distal site		
Baseline	0.72 ± 0.17	0.10 ± 0.04
12-month	1.06 ± 0.20	0.25 ± 0.06
*p*	0.006 **	0.004 **

**Note:** Data presented as the mean ± SEM. Out of 60 implants, 30 were of the IOD group, and 30 were of the FP group. A paired *t*-test was used. *** *p* < 0.001, ** *p* < 0.01, and * *p* < 0.05.

**Table 3:** Marginal bone level alteration: Inter-group analysis of IOD and FP.

Time Period	IOD Group	FP Group	*p*-Value
Average			
Baseline	0.62 ± 0.17	0.08 ± 0.03	0.003 **
12-month	0.91 ± 0.16	0.18 ± 0.04	<0.001 ***
Mesial site			
Baseline	0.52 ± 0.19	0.05 ± 0.02	0.016 *
12-month	0.76 ± 0.15	0.10 ± 0.04	<0.001 ***
Distal site			
Before	0.72 ± 0.17	0.10 ± 0.04	0.001 **
12-month	1.06 ± 0.20	0.25 ± 0.06	<0.001 ***

**Note:** Data presented as the mean ± SEM. Out of 60 implants, 30 were of the IOD group, and 30 were of the FP group. An unpaired *t*-test with Welch’s correction was used. *** *p* < 0.001, ** *p* < 0.01 and * *p* < 0.05.

**Table 4:** The effect of implant parameters on osseointegration and marginal bone level outcomes at the 12-month follow-up in the implant-supported fixed prostheses and implant-retaining overdenture groups according to gender.

Index	Gender	*n*	IOD Group	*n*	FP Group	*p*
**MBL (mm)**	**Male**	17	0.91 ± 0.12	12	0.03 ± 0.02	<0.001 ***
	**Female**	13	0.92 ± 0.35	18	0.28 ± 0.06	0.092
**MBLC (mm)**	**Male**	17	0.34 ± 0.11	12	0.03 ± 0.02	0.015 *
	**Female**	13	0.22 ± 0.07	18	0.15 ± 0.04	0.383
**r-BIIC (%)**	**Male**	17	95.40 ± 0.99	12	99.75 ± 0.25	<0.001 ***
	**Female**	13	97.24 ± 0.53	18	98.50 ± 0.42	0.077

**Note:** Data presented as the mean ± SEM. *n* indicates the number of implants. MBL, marginal bone level; MBLC, marginal bone level change; r-BIIC, radiographic bone-implant interface contact. Out of 60 implants, 30 were of the IOD group, and 30 were of the FP group. An unpaired *t*-test with Welch’s correction was used. *** *p* < 0.001, and * *p* < 0.05.

**Table 5:** The effect of implant parameters on osseointegration and marginal bone level outcomes at the 12-month follow-up in the implant-supported fixed prostheses and overdenture groups according to the lower site.

Index	Location	*n*	IOD Group	*N*	FP Group	*p*
**MBL (mm)**	Lower jaw	23	1.16 ± 0.14	19	0.13 ± 0.04	<0.001 ***
**MBLC (mm)**	Lower jaw	23	0.28 ± 0.09	19	0.12 ± 0.04	0.115
**r-BIIC (%)**	Lower jaw	23	96.08 ± 0.79	19	98.79 ± 0.43	0.005 **

**Note:** Data presented as the mean ± SEM. *n* indicates the number of implants. MBL, marginal bone level; MBLC, marginal bone level change; r-BIIC, radiographic bone-implant interface contact. An unpaired *t*-test with Welch’s correction was used. *** *p* < 0.001, and ** *p* < 0.01.

**Table 6:** The effect of implant parameters on osseointegration and marginal bone level outcomes at the 12-month follow-up in the implant-supported fixed prostheses and overdenture groups according to length.

Index	Length	*n*	IOD Group	*N*	FP Group	*p*
**MBL (mm)**	10 mm	28	0.90 ± 0.17	30	0.18 ± 0.04	<0.001 ***
**MBLC (mm)**	10 mm	28	0.26 ± 0.07	30	0.10 ± 0.03	0.036 *
**r-BIIC (%)**	10 mm	28	96.50 ± 0.54	30	99.00 ± 0.29	<0.001 ***

**Note:** Data presented as the mean ± SEM. *n* indicates the number of implants. MBL, marginal bone level; MBLC, marginal bone level change; r-BIIC, radiographic bone-implant interface contact. An unpaired *t*-test with Welch’s correction was used. *** *p* < 0.001, and * *p* < 0.05.

**Table 7:** The effect of patient and implant parameters on osseointegration and marginal bone level outcomes at the 12-month follow-up in the overdenture group (*n* = 30).

Characteristics	MBL (mm)	*p*	MBLC (mm)	*p*	r-BIIC (%)	*p*
**Gender**						
Male	0.91 ± 0.12	0.970	0.34 ± 0.11	0.391	95.40 ± 0.99	0.115
Female	0.92 ± 0.34		0.22 ± 0.07		97.24 ± 0.53	
**Location**						
Upper jaw	0.09 ± 0.41	0.042 *	0.32 ± 0.09	0.797	96.59 ± 0.72	0.639
Lower jaw	1.16 ± 0.14		0.28 ± 0.09		96.08 ± 0.79	

**Note:** Data presented as the mean ± SEM. *n* indicates the number of implants. The male group had 17 implants; 13 for the female group. Seven implants were placed in the upper jaw, whereas 23 were in the lower jaw group. An unpaired *t*-test with Welch’s correction and a Welch ANOVA test was used. * *p* < 0.05.

**Table 8:** The effect of patient and implant parameters on osseointegration and marginal bone levels at the 12-month follow-up in the implant-supported fixed prostheses group. **(*n* = 30)**.

Parameter	MBL (mm)	*p*	MBLC (mm)	*p*	r-BIIC (%)	*p*
**Gender**						
Male	0.03 ± 0.03	0.001	0.03 ± 0.03	0.018 *	99.75 ± 0.25	0.018 *
Female	0.28 ± 0.06		0.15 ± 0.04		98.50 ± 0.42	
**Location**						
Upper jaw	0.25 ± 0.09	0.293	0.06 ± 0.03	0.272	99.36 ± 0.27	0.272
Lower jaw	0.13 ± 0.04		0.12 ± 0.04		98.79 ± 0.43	
**Diameter**						
4.1 mm	0.13 ± 0.06	0.483	0.13 ± 0.06	0.620	98.71 ± 0.64	0.620
4.8 mm	0.19 ± 0.05		0.09 ± 0.03		99.09 ± 0.33	

**Note:** Data presented as the mean ± SEM. *n* indicates the number of implants. The male group had 12 implants, while 18 for the female group. Eleven implants were placed in the upper jaw, whereas 19 were in the lower jaw group. 7 and 23 implants were 4.1 mm, and 4.8 mm wide, respectively. However, out of 30 implants, 30 implants were 10 mm long. An unpaired *t*-test with Welch’s correction. * *p* < 0.05.

## Data Availability

Data is contained within the article.
